# Respiratory constraints during activities in daily life and the impact on health status in patients with early-stage COPD: a cross-sectional study

**DOI:** 10.1038/npjpcrm.2016.54

**Published:** 2016-10-13

**Authors:** Hanneke AC van Helvoort, Laura M Willems, PN Richard Dekhuijzen, Hieronymus WH van Hees, Yvonne F Heijdra

**Affiliations:** 1 Department of Pulmonary Diseases, Radboud university medical center, Nijmegen, The Netherlands; 2 Department of Pulmonary Diseases, University center for chronic diseases Dekkerswald, Groesbeek, The Netherlands

## Abstract

In patients with chronic obstructive pulmonary disease (COPD), exercise capacity is reduced, resulting over time in physical inactivity and worsened health status. It is unknown whether ventilatory constraints occur during activities of daily life (ADL) in early stages of COPD. The aim of this study was to assess respiratory mechanics during ADL and to study its consequences on dyspnoea, physical activity and health status in early-stage COPD compared with healthy controls. In this cross-sectional study, 39 early-stage COPD patients (mean FEV_1_ 88±s.d. 12% predicted) and 20 controls performed 3 ADL: climbing stairs, vacuum cleaning and displacing groceries in a cupboard. Respiratory mechanics were measured during ADL. Physical activity was measured with accelerometry. Health status was assessed by the Nijmegen Clinical Screening Instrument. Compared with controls, COPD patients had greater ventilatory inefficiency and higher ventilatory requirements during ADL (*P*<0.05). Dyspnoea scores were increased in COPD compared with controls (*P*<0.001). During ADL, >50% of the patients developed dynamic hyperinflation in contrast to 10–35% of the controls. Higher dyspnoea was scored by patients with dynamic hyperinflation. Physical activity was low but comparable between both groups. From the patients, 55–84% experienced mild-to-severe problems in health status compared with 5–25% of the controls. Significant ventilatory constraints already occur in early-stage COPD patients during common ADL and result in increased dyspnoea. Physical activity level is not yet reduced, but many patients already experience limitations in health status. These findings reinforce the importance of early diagnosis of COPD and assessment of more than just spirometry.

## Introduction

Chronic obstructive pulmonary disease (COPD) is characterised by chronic inflammation of the airways and lungs, persistent airflow limitation and, often, a progressive decline in lung function. Nowadays, it is associated with multiple and significant morbidity and increased mortality.^1^ Despite this prospect, COPD remains often undiagnosed and untreated until the disease has progressed to a point where lung function and quality of life are severely affected.^2^

Many had or still have the perception that mild airway obstruction (i.e., post-bronchodilator forced expiratory flow in 1 s (FEV_1_)/forced vital capacity <0.7 and FEV_1_ >80% predicted) has few clinical consequences and does not require intervention. However, there is growing evidence that even mild airway obstruction is associated with a reduction in exercise capacity and physical activity in patients with COPD.^3–6^ Even before patients are aware of their illness, they often evade some of their (more strenuous) activities to avoid unpleasant symptoms, such as dyspnoea. This can, over time, lead to a negative spiral of worsening symptoms, deconditioning and exercise intolerance, as patients become progressively more sedentary.^7,8^

An understanding of the structure–function relationships in the lungs and airways may assist in understanding exercise limitations, also in early stages of COPD. A range of structural abnormalities, including airway wall thickening, pulmonary gas trapping, emphysema and vascular dysfunction,^9–11^ contribute to high ventilatory demands^5,12,13^ and impairment of dynamic respiratory mechanics in patients with COPD. These mechanisms of exercise limitations have all been extended to patients with mild COPD,^5,9,12^ indicating that the respiratory system reaches its physiological limits at the end of (strenuous) exercise. Furthermore, cardiocirculatory impairment^14–16^ and skeletal muscle dysfunction^17–20^ have been reported in mild-to-moderate COPD, and may, in turn, also lead to reduced exercise capacity and activity limitation. However, it remains unclear as to which of all the mentioned mechanisms, and to what extent, actually contributes to respiratory system limitations during activities of daily life(ADL), as these activities are normally much less strenuous than exercise tests.

**Figure 1:**
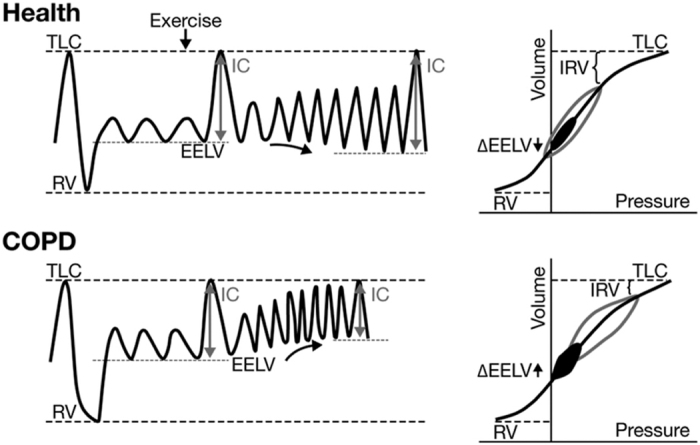
Lung volumes at rest and during exercise in healthy subjects and in patients with chronic obstructive pulmonary disease (COPD). In normal lungs, end-expiratory lung volume (EELV) remains relatively constant during exercise, as tidal volume can increase and inspiratory capacity (IC) is maintained. Patients with COPD breathe with a greater EELV and less IC. During exercise, as ventilation increases, the increased EELV (dynamic hyperinflation) pushes tidal volume closer to total lung capacity (TLC) where expansion is limited by high pressures. IC decreases and breathing becomes so restricted that patients have to stop activity. IRV, inspiratory reserve volume; RV, residual volume. From ref. 30 with permission.

In the current study, respiratory mechanics during different ADL were investigated in COPD patients with relatively preserved airflow, defined here as early-stage COPD (FEV_1_>65% predicted). Healthy control subjects were used for comparison. The aim was to investigate whether changes in respiratory mechanics (i.e., high ventilatory demands and dynamic hyperinflation (Figure 1)) also occur in early-stage COPD during common ADL and thereby result in more dyspnoea. Because of the potential role of respiratory limitations for important clinical outcomes such as dyspnoea, physical activity and health status, it was also cross-sectionally investigated whether these outcomes indeed were affected in early-stage COPD compared with healthy controls. The measurement of respiratory mechanics (i.e., dynamic hyperinflation) especially during ADL makes this study unique and additional to other results presented in the literature in this field.

## Results

### Subjects

General characteristics and pulmonary function data are presented in Table 1. COPD patients did smoke more pack-years and experienced more dyspnoea on the MRC scale compared with healthy controls.

### Physiology and respiratory mechanics during ADL

Table 2 shows the physiological response to all three ADL for patients and controls. Although oxygen uptake was comparable for patients and controls in each activity, ventilation was significantly higher in patients with COPD. In addition, COPD patients had greater ventilator inefficiency compared with controls in all three ADL. BORG dyspnoea scores at end ADL were increased in COPD patients compared with controls. None of the subjects reached ventilation levels higher than 85% of predicted maximal voluntary ventilation during ADL. Mean change in IC was negative in patients but positive in controls. Mean inspiratory reserve volume (IRV) was preserved.

**Figure 2:**
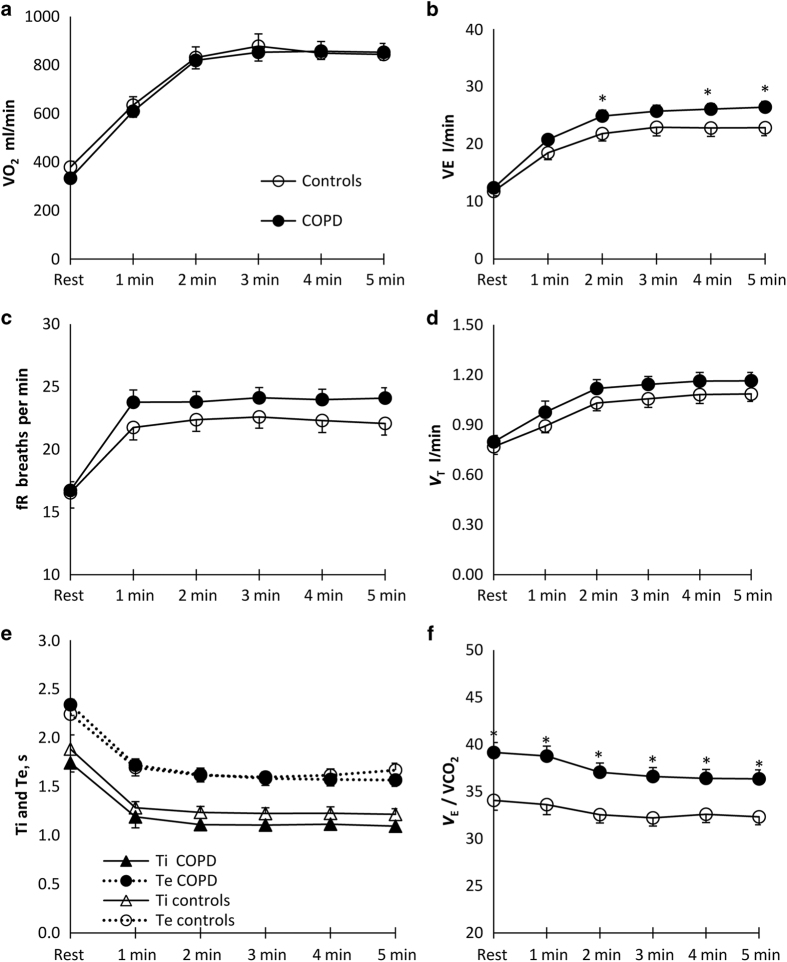
Metabolic and ventilatory responses to vacuum cleaning. (**a**) Oxygen uptake (VO_2_), (**b**) minute ventilation (VE), (**c**) respiratory frequency (fR), (**d**) tidal volume (Vt), (**e**) inspiratory time (Ti) and expiratory time (Te) and (**f**) VE/carbon dioxide production (VCO_2_). Data are presented as mean±s.e.m. *P<0.05 at standardised time points.

Figure 2 shows the metabolic and ventilatory responses to one of the ADL, namely vacuum cleaning.

Compared with healthy controls, COPD patients had greater ventilatory inefficiency (Figure 2f) and higher ventilatory requirements (Figure 2b). The response to climbing stairs and placing groceries in a cupboard was overall comparable (data not shown).

**Figure 3:**
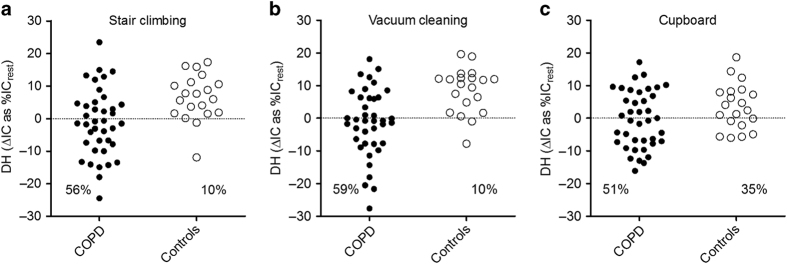
Dynamic hyperinflation (DH), expressed as change in IC, after different ADL. (**a**) Stair climbing, (**b**) vacuum cleaning and (**c**) displacing groceries in a cupboard. Percentages indicate proportion of subjects demonstrating a decrease in IC (hyperinflators).

In Figure 3, individual data of DH at the end of each activity are shown. More than half of the patients with COPD showed DH during climbing stairs, vacuum cleaning and unloading groceries (56%, 59% and 51%, respectively). In contrast, much less controls demonstrated DH (10%, 10% and 35% with *P*=0.002, *P*=0.001 and *P*=0.362, respectively). Thirty patients (77%) had DH in at least one of the activities, compared with only eight (40%) control subjects (*P*=0.012). Fourteen patients (36%) had DH during each ADL, compared with no controls (*P*=0.006).

Within the patient group, no correlation existed between DH during any ADL and the severity of airflow obstruction (expressed as FEV_1_% predicted). In addition, no relation was found between DH during the different ADL and the existence of static hyperinflation at rest (RV/total lung capacity (TLC)). The amount of DH did not show a significant relation with increases in BORG scores after the ADL. However, if the COPD patients were identified as either dynamic hyperinflators (IC decrease during an ADL) or non-dynamic hyperinflators (no IC decrease), the hyperinflators scored higher increases in BORG scores compared with non-hyperinflators after both stair climbing (2.9±1.0 versus 2.0±1.0, *P*=0.03) and vacuum cleaning (1.9±0.8 versus 1.2±0.7, *P*=0.04).

### Physical activity

**Figure 4:**
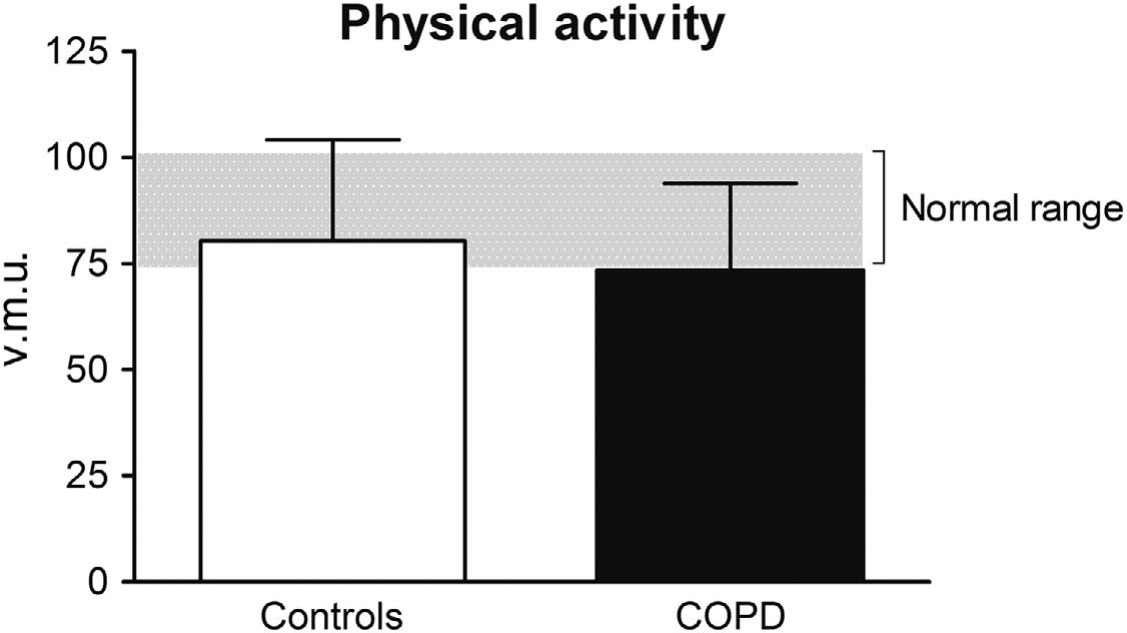
Mean physical activity of healthy controls compared with COPD. No significant differences were seen.

Although the above-mentioned physiological differences in the performance of ADL were found, the total group of patients with COPD did not differ significantly from healthy controls according to physical activity (74±21 vector magnitude units (v.m.u.) versus 80±24 v.m.u., *P*=0.273); see Figure 4. The COPD patients had a mean physical activity similar to the lower limit of normal range (74) for this age group (normal range 74–102).

A decreased FEV_1_% predicted or the occurrence of DH could not be associated with physical inactivity within these study groups.

### Health status

**Figure 5:**
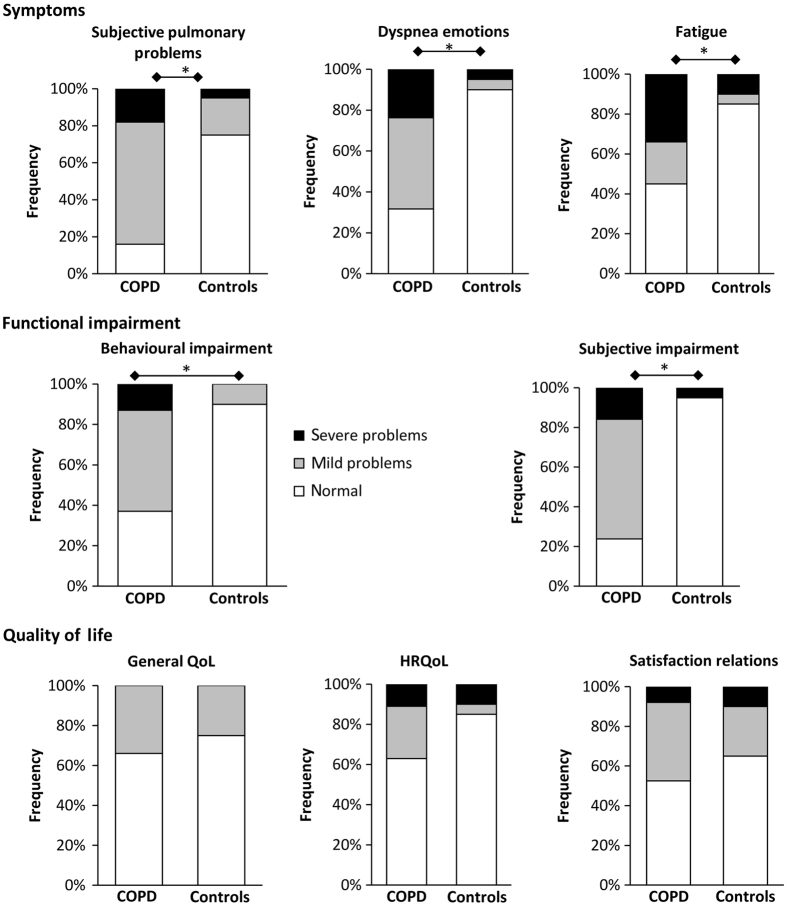
Frequencies of patients and healthy controls experiencing normal functioning (white), mild (grey) or severe (black) problems in each sub-domain of the NCSI. *P<0.05 between groups.

Finally, many of these early-stage COPD patients scored mild or severe problems in the sub-domains of the Nijmegen Clinical Screening Instrument (NCSI). In Figure 5, it is shown that in the domains of Symptoms and Functional Impairments these COPD patients score significantly more mild and severe problems than the healthy controls. No differences in the domain of Quality of Life were observed.

## Discussion

### Main findings

Performing common ADL has been shown to result in a higher ventilatory demand (VE), less ventilatory efficiency (VE/VCO2), frequent occurrence of DH and higher dyspnoea scores in early-stage COPD patients compared with healthy subjects. This group of COPD patients with relatively preserved airflow were overall normal physically active but, already very early in the disease their health status, had declined compared with healthy subjects of the same age. In particular, symptoms (i.e., dyspnoea and fatigue) and functional impairment were already reported as mildly-to-severely affected by about two-thirds of the patients.

### Interpretation of findings in relation to previously published work

Ventilatory requirements have been consistently elevated for different exercise intensities in patients with mild COPD relative to controls.^5,9,12^ The present study demonstrated that even during simple and common ADL, these patients experience a higher ventilatory demand compared with healthy controls. In agreement with other studies on respiratory mechanics in COPD, it is now shown that early-stage COPD patients already breathe with greater ventilatory inefficiency during ADL, as reflected by an increased ventilatory equivalent for CO_2._^4,5,9,12^ Recently, the mechanisms of high V_E_/VCO_2_ in mild COPD and its impact on dyspnoea and exercise intolerance were studied during incremental exercise testing with invasive arterial blood sampling.^4^ The investigators found pulmonary gas-exchange abnormalities (physiological dead space and wasted ventilation) in mild COPD but still found arterial blood–gas homeostasis. They suggested that this arterial blood–gas homeostasis is a result of compensatory increase in minute ventilation. In addition, the higher ventilator demand in the presence of expiratory flow limitation (dynamic hyperinflation at high-intensity exercise) forced earlier mechanical constraints on tidal volume expansion and was associated with an earlier onset of severe dyspnoea in the mild-COPD group compared with controls. Our study used ADL without invasive blood–gas measurements, but these former findings support the early-respiratory changes found in our study, even in ADL. The present study demonstrates for the first time that dynamic hyperinflation occurs during ADL in COPD patients with still relatively preserved airflow. The respiratory phenomenon DH has been described earlier in mild-COPD patients after cardiopulmonary exercise testing (CPET)^5^ or after metronome-paced hyperinflation.^21,22^ Although there was a variety in the amount of DH within the group, our early-stage COPD and also between the different ADL activities, the proportion of hyperinflators in the COPD group was striking. The COPD patients complained about more dyspnoea, which is well-understandable because of the elevated ventilatory demand and inefficiency, and consisted with the earlier mentioned mechanistic study in mild COPD.^4^ However, more importantly, those patients who developed DH during ADL also experienced more dyspnoea compared with the patients who did not develop DH. This indicates a potential role for DH in the development of dyspnoea sensation^23^ during everyday activities, even in this early stage of the disease.

In contrast to the present findings, previous research in patients from the whole GOLD spectrum (I to IV) has shown that the amount of DH is associated with the amount of daily physical activity.^24,25^ There might be several reasons why we were not able to find this. First, physical inactivity was a secondary outcome of the present study, which thus lacks the power to provide significant results. Second, the difference in severity of airflow limitation has an important role in the contrasting results. The majority of the patients of the current study were included from general practice, who still had a normal level of activity, indicating another type of patients compared with the earlier studies.^6^

Quite recently, consensus was obtained about the value of assessment of physical activity and health status in patients with COPD as a clinical outcome in the management of COPD.^26^ A recent European cross-sectional study^27^ showed that patients with mild airflow obstruction already display significant health status impairment. The present study underlines these findings and emphasises that 55–84% of all COPD patients with relatively preserved airflow limitation already experience moderate-to-severe problems on dyspnoea sensation, fatigue and functional impairment because of their COPD. This was in large contrast with only 5–25% of the healthy controls having problems in their health status.

### Strengths and limitations of this study

Because of the observational and cross-sectional characteristics of the current study, no causal relations (in)activity and health status could be studied. However, the physiological agreements between the present findings and other mechanistic studies suggest also similarities in causality of the constraints and their consequences.

Even when included in primary care, it is plausible that patients are diagnosed with their disease based on health problems interfering with health status (as many others are not diagnosed). In other words, a selection bias might have increased the proportion of patients with impaired health status in this study population.

In the study design, it was chosen to study ADL that are commonly performed by the included patients. Hereby, the usual activities and constraints of patients are reflected, which strengthens this study. For the first time, COPD patients with relatively preserved airflow from general practice were studied on breathing mechanics during usual activities. Furthermore, the study also underlined the occurrence of limitations in patient-related outcomes in these early stages of the disease.

### Implications for future research, policy and practice

The effect of treatment on ventilatory constraints in these early-stage COPD patients was beyond the scope of this study. Exploratory studies suggest that pharmacotherapy has beneficial effects on airway physiology in patients with COPD (reviewed in refs 28–31). However, the clinical relevance of these improvements remains to be determined. The current findings again open the discussion whether and when therapy in COPD should be started. Appropriate clinical trials are needed to evaluate the efficacy profile of various pharmacological and non-pharmacological interventions for the management of patients with mild COPD. Next, the natural course of the different ventilatory constraints found in this study remains unclear. An earlier study^21^ suggests that DH worsens at a higher speed than the airflow limitation of COPD patients, indicating the importance of early screening for DH. The current study indicates a potential role for this dynamic respiratory mechanic impairment in the development of dyspnoea sensation during ADL, even in this early stage of the disease. Clearly, prospective studies on DH, its consequences for the patients and eventually the effect of early treatment of DH are needed.

Finally, even patients in these early stages of COPD and with still relatively preserved airflow already decline their activity levels to the lower limit of normal, reflecting the importance of early recognition of the illness and encouragement of physical activity and exercise in mild COPD. Awareness and assessment of physical (in)activity in COPD within general practice deserves high attention.

### Conclusions

The results of this study show elevated ventilatory requirement and ventilatory inefficiency together with dynamic hyperinflation in early-stage COPD patients during the performance of simple and common ADL, resulting in more dyspnoea compared with healthy controls. In addition, patients who develop DH during ADL also experience more dyspnoea than patients without DH, reflecting a potential role of this respiratory limitation in dyspnoea sensation, even in mild COPD patients performing easy ADL. Although the patients from general practices in this study still had a normal physical activity level, a very large proportion of these COPD patients already experienced limitations in health status. These results reinforce the importance of early diagnosis of COPD in general practices and assessment of more than just spirometry. The fact that ventilatory constraints in these mild patients already occur during commonly performed ADL opens again the discussion of early treatment in COPD.

## Materials and methods

### Subjects

Forty patients with stable COPD and relatively preserved airflow (FEV_1_/VC<0.7, FEV_1_⩾65% of predicted after bronchodilation) were included. In this study, patients with an FEV_1_>65 of predicted were included as early-stage COPD patients, and thus reflect the group of GOLD I and the mildest half of GOLD II COPD patients, according to the GOLD initiative.^1^ Patients were recruited from general practices in or close to Nijmegen and from the outpatient population of Radboud university medical center or university center for chronic diseases Dekkerswald. Exclusion criteria were co-existing lung diseases other than COPD (including asthma) and exercise-limiting disorders other than COPD such as cardiac or neuromuscular disease.

In addition, 20 non-obstructive control subjects, aged 40–80 years, without a history of lung diseases were recruited from the same region as the patients. Exclusion criteria for this control group were disorders causing exercise limitation or influencing physical activity (e.g., neuromuscular or skeletal disorders). Healthy subjects who were highly active (performing sport activities >3× a week) were also excluded.

### Study design

Subjects visited our laboratory on 2 days. During the first visit, pulmonary function tests and anthropometrics were performed. Ventilatory and metabolic constraints were measured during the performance of three different ADL. In addition, assessment of physical activity for 7–10 days began. During the second visit (maximal 8 days after visit 1), a CPET was performed and health status was assessed.

This study was conducted in accordance with the amended Declaration of Helsinki and was approved by the local medical ethics committee (CMO Arnhem-Nijmegen, no NL37406.091.11 and NL42423.091.12). All subjects gave their written informed consent.

### ADL measurements

Subjects performed three ADL in a randomised order at their usual pace: climbing stairs (A), vacuum cleaning (B) and placing groceries in a cupboard (C). During ADL-A, subjects climbed 2 floors (39 steps in total). During ADL-B, subjects were asked to vacuum a carpet for 5 min the way they usually do (same position and speed). In addition, subjects displaced 10 products of 1 kg one by one from a ground shelf to a shelf at shoulder height and back (ADL-C). The activities were finished after task completion or until symptom limitation. During the activities, a portable breath-by-breath system (Oxycon Mobile, Jaeger, CareFusion GmbH, Hoechberg, Germany) was used to measure metabolic and ventilatory parameters including inspiratory capacity (IC) to reflect dynamic hyperinflation.^32^ IC manoeuvers were directly visible for the investigators and were performed at rest and directly after each activity sitting upright. Dynamic hyperinflation was expressed as the decrease in IC relative to resting IC (%ΔIC=(IC end−IC rest)/IC rest×100) and/or as absolute change in IC (IC end−IC rest). Shortness of breath was rated using the modified Borg scale (0–10) at the start and end of each ADL.

### Pulmonary function, exercise capacity, physical activity and health status

Spirometry and body plethysmography were performed according to the American Thoracic Society/European Respiratory Society guidelines.^33,34^ Predicted normal values were derived from the European Community for Steel and Coal.^35^ Predicted normal values for IC were calculated as predicted TLC minus predicted functional residual capacity (FRC). Each subject completed a symptom-limited incremental CPET on a cycle ergometer to determine peak oxygen consumption according to the ATS/ACCP guidelines.^36^ Tests consisted of steady-state unloaded cycling for 3 min followed by 5–20 W increase in work rate every minute untill symptom limitation. Subjects cycled on an electrically braked cycle ergometer (Masterlab, Jaeger, Wurzburg, Germany) while breathing through a face mask that was connected to a metabolic system (Oxycon Pro). Measurements included standard breath-by-breath cardiorespiratory and breathing pattern parameters; oxygen saturation by pulse oximetry; heart rate by ECG and dyspnoea intensity assessed with the modified 10-point Borg scale.

To assess daily physical activity, subjects wore a tri-axial accelerometer (Actometer, manufactured by the Department of Electronics and Instrument Services of the Psychological Laboratory of the University of Nijmegen, the Netherlands) at the ankle for at least 7 consecutive days and nights.^37^ Mean daily activity was expressed as v.m.u.

Health status was assessed by the NCSI.^38^

### Statistical analysis

This was an observational physiological study with the main outcome measures consisting of V_E_, V_E_/VCO_2_, VT, Bf and DH. Because of the complex and time-consuming ADL measurements, we planned to include 40 patients and 20 healthy controls. Data of one COPD patient were not reliable and thereby excluded from analyses. In previous studies^5,12,39^ incorporating physiological measurements during ADL and other exercises, this sample size was large enough to detect significant difference in relevant physiological variables.

Baseline characteristics are shown as mean±s.d. or as frequencies. Between-group comparisons of subject characteristics and physiological variables at standardised point during ADL were performed using unpaired Student’s *t*-test. In case of unequal variances or distributions (pack-years, MRC, ΔIC and physical activity), Mann–Whitney tests were used. To test differences in occurrence of dynamic hyperinflation and other categorical variables between patients and controls, the *Χ*^2^-test was used. Associations between lung function, ADL physiology, dyspnoea and physical activity were determined using Spearman's correlations.

*P*<0.05 was indicated as statistically significant. Data were analysed with SPSS 20.0 (IBM Corp, Armonk, NY, USA).

## Figures and Tables

**Table 1:** Subject characteristics, lung function and pulmonary medication

	*Controls (*n*=20)*	*COPD (*n*=39)*
Age, years	62±8	64±8
Gender, m/f	10/10	25/14
BMI, kg/m^2^	25.9±4.9	27.4±5.2
Pack-years, years	6±9	25±18^***^
Smoking status (% never/ex/current)	55/35/10	0/85/15^***^
MRC, 0/1/2/3	12/8/0/0	3/18/16/2^***^
FEV_1_, l^a^	3.25±0.85	2.58±0.57^**^
FEV_1_, %pred	112±15	88±12^***^
FEV_1_/VC, %^a^	75±4	58±7^***^
TLC, %pred	109±11	116±13*
RV/TLC, %	37±8	41±6*
		
*Medication use, % of patients*		
SABA	0%	23%
SAMA	0%	3%
LAMA	0%	46%
LABA	0%	5%
ICS	0%	8%
Combined ICS/LABA	0%	31%
No inhalation medication	100%	28%

Abbreviations: BMI, body mass index; f, female; FEV_1_, forced expiratory volume in 1 s; ICS, inhaled corticosteroid; LABA, long-acting β_2_-agonist; LAMA, long-acting muscarinic antagonist; m, male; pred, predicted; RV, residual volume; SABA, short-acting β_2_-agonist; SAMA, short-acting muscarinic antagonist; TLC, total lung capacity; VC, vital capacity.

a post-bronchodilator values for patients with COPD.

*P<0.05, **P<0.01, ***P<0.001 compared with controls.

**Table 2:** Physiological responses to ADL

	*Climbing stairs*		*Vacuum cleaning*		*Cupboard*	
	*COPD*	*Controls*	*COPD*	*Controls*	*COPD*	*Controls*
VO_2_, l/min	1.3±0.3	1.4±0.3	0.9±0.2	0.9±0.2	1.3±0.3	1.3±0.2
VO_2_, %peak	71±15	67±16	47±13	40±12	69±15	62±16
VE, l/min	37±7*	31±8	27±6*	23±6	36±8*	29±6
VE, %MVV	39±11***	26±9	28±7***	20±7	37±10***	25±8
Bf, per min	24±6	21±4	24±5	23±4	24±6*	21±6
Vt, l	1.65±0.40	1.55±0.28	1.17±0.32	1.06±0.21	1.61±0.56	1.64±0.49
V_E_/VCO_2_	35±6^**^	30±4	36±6**	32±4	30±4	35±5**
IRV, l	1.54±0.59	1.62±0.60	2.00±0.67	2.07±0.65	1.56±0.54	1.37±0.54
ΔIC, l	−0.06±0.31**	0.18±0.20	−0.06±0.33**	0.25±0.23	−0.04±0.29	0.09±0.05
ΔIC, %IC_rest_	−1.2±10.3**	6.5±7.0	−1.4±10.4**	8.6±6.9	−0.5±8.7	3.6±7.0
BORG, dyspnoea	2.6±1.5***	0.8±1.4	1.5±1.4***	0.5±0.7	2.3±1.5***	0.6±0.8
Time, s	46±9	43±6	300±0	300±0	81±13**	70±14

Abbreviations: Bf, breathing frequency; IC, inspiratory capacity; IRV, inspiratory reserve volume; MVV, maximal voluntary ventilation; VE, ventilation; VO_2_, oxygen consumption; Vt, tidal volume.

*P<0.05 compared with controls; **P<0.01 compared with controls; ***P<0.001 compared with controls.
